# Chronological Review and Rational and Future Prospects of Cannabis-Based Drug Development

**DOI:** 10.3390/molecules25204821

**Published:** 2020-10-20

**Authors:** Dvora Namdar, Omer Anis, Patrick Poulin, Hinanit Koltai

**Affiliations:** 1 Institute of Plant Science, Agriculture Research Organization, Volcani Center, Rishon LeZion 7528809, Israel; dvoran@volcani.agri.gov.il; 2 Department of Urology, Sheba Medical Center, Ramat Gan 5262000, Israel; omerwn@gmail.com; 3 Consultant Patrick Poulin Inc., Québec City, QC G1V 0A6, Canada; patrick.poulin.4@umontreal.ca; 4 School of Public Health, Université de Montréal, Montréal, QC H3T 1J4, Canada

**Keywords:** cannabis, cannabinergic, drug, FDA-approved, medical conditions, pharmaceutical-grade, phytocannabinoid

## Abstract

Despite the surge in cannabis chemistry research and its biological and medical activity, only a few cannabis-based pharmaceutical-grade drugs have been developed and marketed to date. Not many of these drugs are Food and Drug Administration (FDA)-approved, and some are still going through regulation processes. Active compounds including cannabinergic compounds (i.e., molecules targeted to modulate the endocannabinoid system) or phytocannabinoid analogues (cannabinoids produced by the plant) may be developed into single-molecule drugs. However, since in many cases treatment with whole-plant extract (whether as a solvent extraction, galenic preparation, or crude oil) is preferred over treatment with a single purified molecule, some more recently developed cannabis-derived drugs contain several molecules. Different combinations of active plant ingredients (API) from cannabis with proven synergies may be identified and developed as drugs to treat different medical conditions. However, possible negative effects between cannabis compounds should also be considered, as well as the effect of the cannabis treatment on the endocannabinoid system. FDA registration of single, few, or multiple molecules as drugs is a challenging process, and certain considerations that should be reviewed in this process, including issues of drug–drug interactions, are also discussed here.

## 1. Introduction

### 1.1. Motivation

This review aims to explore the chronology, as well as rational and future prospects, of cannabis-based drug development. The chronological path toward the development of cannabis-based drugs reflects the understanding and accumulating knowledge regarding active compounds and their mode of action. Translating these values into modern, registered, pharmaceutical drugs is a highly challenging task. For the scientific discussion below (see Appendix A for methodology), we chose to include only peer-reviewed articles. In addition, we emphasize in this review cannabis drugs that have gone through legislation processes and registration as approved cannabis-related drugs. We use this list of drugs and scientific discussion to unveil the challenges and milestones that led us to the current situation in this arena, and then we further use this platform to draw outlines for future endeavors in this challenging field.

### 1.2. Basic Cannabis Chemotaxonomy

To date, only a few cannabis-based pharmaceutical grade medicines have been developed and marketed. A review of the market reveals that registered cannabis-based drugs follow the common, rather dated, dichotomous chemo-variation approach to *Cannabis sativa*, where only the relative contents of Δ9-tetrahydrocannabinol (THC) and cannabidiol (CBD) are considered [1]. Under this rather simplistic chemotaxonomy, cannabis strains are divided into three main chemovars on the basis of the relative amounts of the two predominant phytocannabinoids (i.e., cannabinoids produced by the plant). These include chemovar I with high levels of THC (THC >50% of total phytocannabinoid content), the intermediate chemovar II strains with equal relative amounts of THC and CBD, and chemovar III strains producing CBD as the dominant phytocannabinoid [1]. The basic cannabis chemotaxonomy gave grounds to the first drugs that were FDA-registered, as discussed below.

### 1.3. Brief Introduction to the Endocannabinoid System

The endocannabinoid system includes the cannabinoid receptor type 1 (CB1) and cannabinoid receptor type 2 (CB2), their endogenous endocannabinoids ligands (*N*-arachidonoylethanolamine (anandamide) and 2-arachidonoylglycerol (2-AG), and the endocannabinoid metabolism enzymes [2]. The latter include *sn*-1-specific diacylglycerol lipase-α (DGLα), DGLβ, *N*-acyl-phosphatidylethanolaminehydrolysing phospholipase D (NAPE-PLD), monoacylglycerol lipase (MAGL; also known as MGL), and fatty-acid amide hydrolase 1 (FAAH) [3]. The CB1 and CB2 receptors are G-protein-coupled receptors (GPCRs). CB1 is highly abundant in the brain, whereas CB2 is expressed mainly in immune cells but is also present at low levels in neuronal and non-neuronal brain cells [3]. CB1 and CB2 signaling involves alterations in Cyclic adenosine monophosphate (cAMP) levels, Extracellular regulated kinase 1/2 (ERK1/2) phosphorylation, and β-arrestin-2 recruitment [2]. They can also form heterodimers and lead to a biased agonistic response [4]. Alterations in concentrations of the endogenous endocannabinoid ligands, receptor expression or activity, or endocannabinoid metabolic enzyme activity have been found to be associated with varied pathological conditions [3].

### 1.4. Cannabinergic Compounds

Any molecule that modulates the endocannabinoid system, regardless of its chemical structure or pharmacological activity, is termed “cannabinergic” [5]. The cannabinergic group of compounds includes CB1 or CB2 receptor ligands or blockers, substrates or inhibitors of fatty-acid amide hydrolase, and the endocannabinoid transporters. Studies of structure–activity relationships between endocannabinoid receptors and many synthesized cannabinergic compounds (e.g., phytocannabinoid analogues and derivatives) have been performed, along with the determination of cannabinergic biological activities and pharmacokinetic properties [6,7,8]. Cannabinergic compounds are considered as targets for the development of novel medications, e.g., for pain management [7,9] or as anti-inflammatory therapeutics [10].

## 2. Chronology of Cannabis-Based Drug Development

### 2.1. Drugs of One Molecule

#### 2.1.1. Cannabis-“Inspired” Drugs

Using a synthetic compound over the purified phytocannabinoid reflects the desire to brand a drug as a non-plant material, yet “inspired” by plant compounds. The first registered drug, Marinol^®^, contains high amounts of dronabinol, the synthetic equivalent of THC (Table 1). Marinol was registered and clinically tested for appetite stimulation and as an antiemetic. The antiemetic efficacy of Marinol was greatest in patients receiving cytotoxic therapy for Hodgkin’s and non-Hodgkin’s lymphomas. 

#### 2.1.2. Cannabis-Derived Drugs

The second drug registered, Epidiolex^®^, contains high amounts of plant-derived CBD (Table 1). Epidiolex, in development since 2002, was the first cannabis-derived drug approved by the United States US Food and Drug Administration (FDA) in 2018. The FDA granted approval of Epidiolex for the treatment of two rare and severe types of epilepsy—Dravet syndrome and Lennox–Gastaut [11,12]. Other single-molecule drugs, inspired by or based on cannabis, were approved for the treatment of different medical conditions including cancer-related pain relief, appetite stimulation, and nausea and vomiting associated with cancer chemotherapy (Table 1). Recently, the FDA approved Epidiolex for the treatment of seizures associated with tuberous sclerosis complex (TSC) in patients 1 year of age and older [13]. Pure CBD has been shown in multiple preclinical and clinical studies to benefit Alzheimer’s disease (AD) and multiple sclerosis (MS) patients. CBD was also shown in preclinical and clinical studies to have antiepileptic activity and was suggested to benefit Parkinson’s disease (PD) patients. However, more data are needed on the benefits of CBD activity for AD, MS, and PD patients [14]. It should be noted that CBD usage carries some adverse side effects. CBD use in animal models led to developmental toxicity, whereas, in human models, CBD use was associated with several adverse effects including diarrhea, fatigue, vomiting, somnolence, and certain drug–drug interactions (detailed below) [15]. CBD toxicity and adverse effects should be stressed in light of the various marketing campaigns and anecdotal reports on the beneficial effects of CBD on multiple medical conditions, as well as the unregulated and ready availability of CBD products in some part of the world.

### 2.2. Drugs of Combinatoric Formulations

Sativex, a GW Pharmaceuticals drug designated for pain relief with relative equal amounts of both THC and CBD and some terpenes (Table 1), marked the updated notion of the power of plant-derived drugs based on the synergistic effect of different components. Sativex was targeted for the treatment of moderate to severe spasticity related with multiple sclerosis (MS). This is specifically in patients who have not responded sufficiently to other anti-spasticity medication. The results of a pivotal phase 3 trial suggested a successful Sativex therapy for this indication [22]. In addition, Sativex was shown to be effective for the treatment of other medical conditions. For example, treatment with Sativex was demonstrated to markedly improve the frequency and severity of motor and vocal tics post treatment in treatment-resistant Tourette syndrome patients [23]. Sativex is not yet approved by the FDA but it is registered for commercial distribution in Europe and Canada.

## 3. Taking Advantage of the “Entourage Effect”

For many centuries, plants and plant extracts were used as phytomedicines for therapeutic treatments for a vast variety of symptoms and medical conditions [24]. Phytomedicines are based on the medical activity of active compounds present in plants. Moreover, in many cases, the biological effect of the whole-plant extract (solvent extraction, galenic preparation, or crude oil) was shown to have preferred activity over treatment with a single purified molecule. The enhancement of activity detected in phytomedicines was designated as the “entourage” effect [25]. As detailed above, the idea behind drug development based on phytomedicines, like that of Marinol of GW pharmaceuticals, was to obtain the most abundant active compounds present in cannabis inflorescence and to mimic their activity using purified compounds administered in known dosages. However, different components present in plant extracts often promote the activity of the lead active compound(s) [26]. Hence, the ability of the “entourage” effect to enhance medical activity should be examined.

One major difficulty with evaluating the entourage effect of traditional medicines is that the underlying mechanism of action is unresolved. In fact, traditional medicines in general and phytomedicines in particular assume this “entourage” as a concept or philosophy of therapy and, in many cases, strive to achieve it using a single-plant extract or some mixture of multiple plants. Some of the effects of compounds present in a given herbal preparation may be enhanced due to the cumulative activity of its constituents. In many other cases, the enhancement of activity by the combination of compounds may be at a synergistic level, as paired combinations of compounds exert effects that are more than the sum of their separate effects [27]. Synergy may be based on enhanced bioavailability and ease of transport of active compounds across barriers, such as cell or organelle membranes, or enhanced protection of an active molecule from degradation by enzymes [28]. Synergy may also result from the activation of more than one signaling pathway in the host cells, leading to an increased response [29].

The “entourage effect” as the enhanced activity of combinations of phytocannabinoids was first recognized by Mechoulam and Ben-Shabat [30]. We suggested that the “entourage effect” was one of the main motivations and considerations in the domestication of cannabis [31]. Synergy in cannabis was recently demonstrated between phytocannabinoids [32,33] and further between phytocannabinoids and terpenes [34]. Moreover, insight into the synergy between cannabis compounds was provided at the levels of both chemical composition and respective biological activity [33,35]. The identified synergistic interactions between cannabis molecules might be the “entourage effect” reported for cannabis preparations, as synergistic effects activate new biological pathways not activated by the components in isolation [33,35]. Importantly, herbal preparations of cannabis, cannabis varieties for use in medical preparations, and guidelines for prescribing doctors including physician’s recommendations, dosages, and titration strategies were recently reviewed in [36]. However, the incomplete understanding (for now) of the mechanism behind this “entourage effect” in cannabis preparations makes it difficult to follow regular drug development and approval procedures (discussed below).

Therefore, we suggest that the selection of combinations of highly active plant ingredients (APIs) from cannabis may open up new opportunities for drug development to treat various medical conditions [31]. Identifying the APIs and their specific compositions within a plant extract, followed by the manufacture of a pure and quantifiable compositions, may lead to pharmaceutical drugs inspired by or based on only the beneficial compounds in cannabis for a certain medical condition. Importantly, these might be targeted to specific mechanisms involved with different diseases [31,33,37].

## 4. Other “Entourage” Considerations

### 4.1. The “Parasitage Effect”

While the overwhelming richness of compounds in cannabis strains can enhance activity, they can also depress activity. In addition to the APIs for the treatment of a given condition, whole extracts (solvent extraction, galenic preparation, or crude oil) also contain other compounds which do not contribute to the desired biological or clinical effect. Moreover, in cases where activity is improved by selecting the most active fractions and eliminating parts of the whole extract, there is a general implication that there might also be negative molecular interactions. Indeed, it was found that the removal of antagonistic or nonactive compounds lowered the required concentrations of APIs [33]. We call this phenomenon the “parasitage effect”; in physics, the term “parasitage” (*French*) describes destructive interference resulting from the interaction of coherent waves (for example) coming from the same source [38]. We use this term here to describe the contra-“entourage effect”, a phenomenon in which certain coproduced compounds interfere with each other to diminish a chemo-biologic effect, instead of correlating to enhance it.

The “parasitage effect” of negative molecular interactions might result from the blockage of API-activated pathways. Cannabinoids target multiple receptors (e.g., CB1, CB2, transient receptor potential vanilloid receptor 1 (TRPV1)) [39]. Hence, antagonistic interference with API receptor binding might also provide evidence for the existence of the parasitage effect. Yet, specific negative interactions between cannabis compounds should still be demonstrated and the mechanism explored.

### 4.2. Interaction between Endocannabinoids and Cannabinoids (Synthetic or Plant-Derived)

Some of the effects of phytocannabinoids involve modulation (activation/inhibition) of receptors and enzymes associated with the endocannabinoid system, which may suggest that at least part of the phytocannabinoid mode of action involves modulation of endocannabinoid activity [40,41]. For example, THC and its propyl synthetic analogue tetrahydrocannabivarin (THCV) are capable of binding with high affinity to CB1 and CB2 receptors [39]. In another example, THC and non-THC phytocannabinoids activate and desensitize human heat-sensitive TRP channels of vanilloid type-1 (TRPV1). Anandamide is an endogenous agonist of TRPV1 [40]. Since both endocannabinoids and phytocannabinoids activate the same receptor (TRPV1 in this case), phytocannabinoids may share activity exhibited by endocannabinoids acting via the endocannabinoid receptors [40]. Phytocannabinoids may also alter endocannabinoid levels; CBD was shown to stimulate TRPV1 and, thus, inhibit anandamide uptake, leading to increased levels of endogenous AEA in an in vitro study [42]. Phytocannabinoids were also shown to alter the activity of endocannabinoid enzymes; CBD in vivo treatment of the nude mouse xenograft model increased the activity of FAAH while decreasing anandamide content in tumor samples [43]. However, the relevance of the interactions between phytocannabinoids and the endocannabinoid system to clinical uses needs to be further studied. Regardless, the effect on the endocannabinoid system by cannabis-inspired or cannabis-based drugs formulated from one or multiple molecules should be considered.

### 4.3. Degradation and Loss of Activation

Natural ratios of phytocannabinoids have been used as the basis for drug development. Several ratios found in plants display higher activity than others [34]. When these phytocannabinoid ratios are altered, there is no enhancement of activity [44]. Additionally, certain phytocannabinoids are more susceptive to degradation and more volatile than others [45,46]. Good manufacturing practices (GMP) should take into consideration the degradation of phytocannabinoids in medical-grade products to maintain the effective natural or established ratios and avoid substantial reduction in activity.

## 5. Drug–Drug Interactions

A change in a drug’s effect on the body when taken together with other drugs, such as common drug–drug interactions (DDIs), should be considered more broadly since DDI may modulate efficacy versus toxicity. Most commonly, DDI results in adverse drug events, which may be caused from changes in pharmaceutical, pharmacokinetic, or pharmacodynamic properties. The interactions between plant-derived products and synthetic drugs (herein “drugs”) are based on the same pharmacokinetic and pharmacodynamic principles as DDIs. The studies of drug–drug, food–drug, and particularly plant-derived drug–drug interactions and genetic factors affecting pharmacokinetics and pharmacodynamics are expected to improve drug safety and to enable personalized drug therapy. Attention is needed for interactions between plant-derived products and synthetic drugs (or naturally occurring chemicals) with a narrow therapeutic index (i.e., a narrow range of doses that confer effectivity without unacceptable adverse effects). However, to date, well-designed clinical studies evaluating herbal supplement–drug interactions are limited; however, case reports are available [47,48,49].

On the other hand, there are numerous in vitro and in vivo studies indicating that phytocannabinoid metabolism is mostly performed by the cytochrome P450 (CYP) isoenzymes. For example, CBD is metabolized by CYP 3A4 and 2C19 and to a lesser extent by CYP 2c8, 2C9, 1A2, 2C8, 2B6, and 2E1 [50,51]. This metabolism via CYP enzymes may inhibit or enhance their activity and, thus, affect the metabolism of various drugs, resulting in different DDI outcomes. In one case study, a threefold increase in tacrolimus levels was detected following the addition of CBD due to CYP3A4/5 inhibition. CBD also inhibits CYP2C19, and a threefold increase in levels of the active metabolite of clobazam has been reported [52]. Interactions with other drugs metabolized by these isoenzymes should be anticipated.

In addition, CBD is a potent inhibitor of CYP2D6 [53]. CYP2D6 metabolizes many antidepressants; thus, CBD may increase serum concentrations of selective serotonin reuptake inhibitors (SSRIs), tricyclic antidepressants, antipsychotics, beta blockers, and opioids (including codeine and oxycodone) [54]. In contrast, any drug presenting an inhibiting or inducing effect on one of these enzymes may change CBD pharmacodynamics and, thus, affect CBD levels [55]. These effects should be considered in the risk–benefit assessment of CBD therapy, and patients and consumers should be made aware of the potential safety hazards of CBD use. Some CYP isoform activity is crucial for the endocannabinoid system. Cardiac CYP2J2 for example, which is inhibited by several phytocannabinoids, results in reduced metabolism of endogenous cardioprotective cannabinoids, such as anandamide [56].

Overall, although further research on DDIs of CBD is needed, CBD may have serious interactions with drugs. It can affect the levels of other drugs, while its own level can be increased or decreased by other medications, and additive effects of CBD can occur with other drugs (e.g., in the case of painkillers).

## 6. Registration Drugs: One Molecule or Combinatoric Formulations

There is a significant interest in the development of therapies and other consumer products derived from cannabis and its components, including CBD. Registering one, a few, or multiple molecules as drugs is a challenging task. Despite the synergistic effect of plant extracts over single compounds, traditional medicines based on whole plants, herbal extracts, or mixtures cannot be recognized as certified drugs as they are non-registerable [36,57]. Registerable drugs are based on known amounts of pure compounds, which can be synthesized and recombined and, thus, registered as formulated medicines. Chemical compositions of plant extracts are highly complex with up to hundreds of different components. Thus, their exact combination is not reproducible by plant growth according to the good manufacturing practices (GMP). Therefore, identifying active compounds in order to either purify or synthesize the determined active ingredients into effective and reproducible drugs is the focus of pharmaceutical companies.

Obviously, patients should have confidence in the drug’s uniformity, strength, and consistent delivery that support appropriate dosing needed to treat patients with complex and serious conditions. This also applies to “combination products”. To that purpose, the FDA issued guidance for GMP requirements for combination products [58], to codify the availability and quality of drugs, biologics, devices, and combination products that consistently meet applicable requirements and specifications both as combination products or as single drugs. Reasons for the rejection of combination products might include pharmacokinetics and/or pharmacodynamic interactions between the combined constituents, the effects of additional manufacturing steps, or other differences arising from combinations.

An FDA guideline was also issued on the regulation of cannabis and cannabis-derived products, including CBD. The FDA stated that it recognizes the potential opportunities that cannabis or cannabis-derived compounds may offer and acknowledges the significance of these possibilities. However, it is also aware of some companies that are marketing cannabis-derived compounds in violation of the Federal Food, Drug, and Cosmetic Act (FD&C Act), and this marketing may put the health and safety of consumers at risk [59].

The drug review and approval process generally contains several defined steps. Table 2 provides a brief summary of the Canadian Drug Review and Approval Process in accordance with the Food and Drugs Act (FDA), the Food and Drug Regulations (FDR), the related policies, and Health Canada guidelines, which are similar in most countries [60]. Canada is the first G20 country to legalize cannabis on a national scale, for both recreational and medicinal purposes. Consequently, several biotech and big pharmaceutical companies have officially entered the Canadian cannabis industry to facilitate cannabis legalization and regulation in other countries.

## 7. Conclusions and Future Prospects

Cannabis-based therapy is a powerful and promising tool as it takes advantage of an existing receptor system to affect multiple body systems and biological processes. However, as this sensitive and balanced endocannabinoids system serves other endogenous molecules and is also associated with drug metabolism, cannabis-based therapy must be carefully examined and applied.

**Figure 1:**
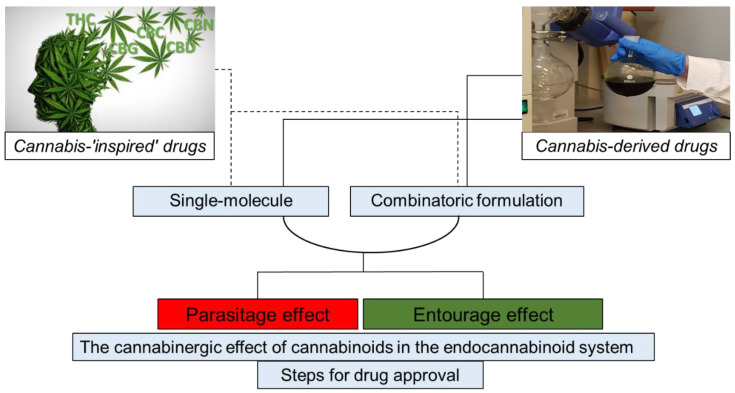
Illustration of concepts associated with the development of cannabis-related pharmaceutical drugs. Cannabis-inspired drugs, cannabinergic drugs, or cannabis-based drugs are to be developed while interaction(s) between phytocannabinoids or synthetic cannabinoids and the endocannabinoid system should be examined. Drug review and approval process steps should be taken.

As illustrated in Figure 1, cannabis-inspired drugs may be developed as synthetic cannabinoids or their derivatives. Cannabinergic drugs may also be developed to specifically attenuate components of the endocannabinoid system (e.g., endocannabinoid receptors). Cannabis-based drugs may be composed of one phytomolecule or as a combination of APIs from the plant. The activity of API combinations may be enhanced by taking advantage of the “entourage” and avoiding the “parasitage” effects. In any case, interaction(s) between endocannabinoids and cannabinoids (synthetic or plant-derived) or cannabinergic compounds must be examined, and steps for drug review should be taken and approval processes be followed, taking into consideration the need to register and approve drugs of combinatoric formulations.

As for the future, we appreciate the aspects involved in cannabis-based drug development, such as complex entourage effects and terpene considerations [33,34,35,36,37]. We also acknowledge the current difficulties of the pharma industry to include natural preparations, as discussed above. However, significant efforts are ongoing in different research laboratories toward revealing the mechanisms and modes of action of other chemical constituents chaperoning the various cannabinoid(s) in whole extracts, such as terpenes and flavonoids. These attempts currently follow indications proved only at the cellular/tissue level. We believe that, as these attempts will bear fruit and the mode of action of the cannabinoids chaperons will be revealed, the pharmaceutical industry will follow these leads for the development of new generations of cannabis-based pharmaceutical products.

## Figures and Tables

**Table 1:** Cannabis-inspired or -based medicines available today and their pharmaceutical status. FDA, Food and Drug Administration; THC, Δ9-tetrahydrocannabinol; CBD, cannabidiol; CBN, cannabinol; CYP, Cytochrome P450; EU, European Union; UGT, uridine 5′ diphospho-glucuronosyltransferase.

Drug Name	Indication	Basic Formulation	Clinical Studies	Proved Efficacy	Key Limiting Toxicity	Recommended Dosage	Drug–Drug Interactions Reported	Approval	Reference
Drugs of one molecule									
MARINOL^®^ (GW Pharmaceuticals, Cambridge, UK)	Appetite stimulation; antiemetic associated with cancer chemotherapy	Dronabinol (synthetic THC)	Yes	Yes	100 mg/day or 30 mg/kg	Appetite stimulation: 2.5 mg twice daily; antiemetic: 5 mg 3–4 times daily		FDA	[16]
EPIDIOLEX^®^ (GW Pharmaceuticals)	Lennox–Gastaut syndrome and Dravet syndrome in patients	Plant derived CBD	Yes	Yes	20 mg/kg/day	5–20 mg/kg/day	CYP1A2, CYP2B6 substrates, uridine 5’ diphospho-glucuronosyltransferase 1A9 (UGT1A9) and UGT2B7. CYP2C8 and CYP2C9 substrates	FDA	[17]
SYNDROS^®^ (Benuvia Therapeutics Inc, Chandler, AZ, USA)	Anorexia; nausea and vomiting associated with cancer chemotherapy	Dronabinol (synthetic THC)	Yes	Yes	25 mg/day	4.2 mg/day	Neuropsychiatric adverse reactions; hemodynamic instability	FDA	[18]
CESAMET^®^ (Valeant Pharma Int, Laval, QC, Canada)	Nausea and vomiting induced by cancer chemotherapy	Nabilone (synthetic THC)	Yes			2 mg/day	Diazepam 5 mg; sodium secobarbital 100 mg; alcohol (absolute) 45 mL; codeine 65 mg	FDA	[19]
INM-755 cream (Inmed pharma, Vancouver, BC, Canada)	Skin diseases and wounds; epidermolysis bullosa	Bacteria *Escherichia coli* fermentation derived from one rare CBN (Cannabinol)	Yes Phase 1–2 ongoing	No		Two doses of INM-755 cream are currently being tested			[20]
Drugs of combinatoric formulations									
SATIVEX^®^ Oromucosal spray (GW Pharmaceuticals)	Pain relief	Plant-derived CBD:THC and terpenes	Yes	Yes	90 mg/day	5–60 mg/day	Reversible inhibitor of CYP3A4, 1A2, 2B6, 2C9, and 2C19	EU, Canada	[21]

**Table 2:** The general drug review and approval process steps. DDIs, drug–drug interactions.

Stage	Activities
1. Initial drug research	Discovering and identifying various chemical and biological substances or other products on the way toward developing a drug; testing for activity, efficacy, toxicity, and ultimately gathering preliminary information on its effectiveness and safety. If the results are promising, researchers proceed to the next stage of development.
2. Preclinical studies	Administration of the drug to selected species of animals (in vivo) or cells (in vitro). The drug must be shown to cause no serious harm (toxicity) at the doses required to have an effect either in a single compound or in DDIs. If results from these initial studies are promising and further tests show acceptable safety levels and clear or potential efficacy, then the next step would be to submit a clinical trial application.
3. Clinical trials	The results of clinical trials conducted with humans are key components of the review process by the regulatory agency. The purpose of a trial is to gather clinical information about a drug’s effectiveness and safety, determine best dosing/usage in humans, evaluate any adverse drug reactions and DDIs, and compare results to already existing treatments for the same disease or condition or to placebo when no treatment already exists for the aimed pathology (when ethically possible). The information gathered from these trials is then included in the dossiers to be reviewed by the relevant agencies.
4. Drug approval process	If results of all the preclinical studies and the clinical trials show that a drug’s potential therapeutic benefit outweighs its risks (side effects, toxicity, etc.), and the chemistry and manufacturing dossier is complete, then the sponsor may decide to file a new drug submission (NDS) with the appropriate regulatory agency in order to be granted authorization to sell the drug in the country.
5. After approval	The regulatory agency requires a sponsor to ensure that the use of its drug is done under the terms of its market authorization. In addition, life cycle management activities (post-approval submissions, for new indications, new dosage forms, new strengths, manufacturing changes, etc.) are required to ensure the maintenance of the product license with its related improvements. In summary, sponsors need to ensure its continued compliance with the food and drug regulations while their products are on the market. On the other hand, the regulatory agency monitors drug information and adverse drug reaction reporting, conducts market surveillance, investigates complaints, and manages recalls if necessary, amongst other things.
6. Additional regulations	There are also more processes and regulations to follow and consider before, during, or after the review process, and before the drug is officially marketed, distributed, and sold in a country. Topics included licensing, warehousing, wholesale distribution rules, and the Drug Establishment License (DEL), as well as regulations around distribution to consumers, regulations around the marketing and advertising activities, provincial requirements, and health insurance funding rules, among others.

## Appendix A

Methodology: To reflect the challenging processes of translation of the accumulating knowledge regarding the active compounds and their mode of action into modern, registered, pharmaceutical drugs, a literature search was done using the following terms: “cannabis”, “*Cannabis sativa*”, “*C. sativa*”, “cannabinoids”, “terpenes”, “medical use”, “herbal preparations”, “hemp”, “oil”, “therapy”, “adverse effects”, “cannabis chemotaxonomy”, “endocannabinoid”, “cannabinergic compounds”, “entourage effect”, “parasitage”, “degradation”, “drug–drug interactions”, and “drug registration”. The search was conducted on general and multidisciplinary research databases for peer-reviewed scientific manuscripts, such as PubMed, Google Scholar, Scopus, and Web of Science. Furthermore, a web search was carried out using the following websites for a non-exhaustive list of examples of medicines: US FDA, Canada’s Food and Drugs Act, and The Drug Review and Approval Processes in Canada (updated to October 2020).
